# A rare case of pheochromocytoma in a pregnant woman presenting with chest pain: extraordinary management

**DOI:** 10.1186/s12872-024-03943-7

**Published:** 2024-05-20

**Authors:** Tao Ge, Xiangrong Xie, Jichun Liu

**Affiliations:** Department of Cardiology, The First Affiliated Hospital of Wannan Medical College, No. 2, Zhe Shan West Road, Wuhu, 241001 Anhui China

**Keywords:** Pheochromocytoma, Angina, Extracorporeal membrane oxygenation, Pregnancy, Coronary angiography

## Abstract

**Background:**

Pheochromocytoma is rare in pregnant women. It presents as diverse symptoms, including hypertension and sweating. The symptoms of pregnant women with pheochromocytoma and comorbid hypertension often mimic the clinical manifestations of preeclampsia, and these women are often misdiagnosed with preeclampsia.

**Case presentation:**

In this case, a pregnant woman presented with chest pain as the primary symptom, and a diagnosis of pheochromocytoma was considered after ruling out myocardial ischemia and aortic dissection with the relevant diagnostic tools. This patient then underwent successful surgical resection using a nontraditional management approach, which resulted in a positive clinical outcome.

**Conclusions:**

It is essential to consider pheochromocytoma as a potential cause of chest pain and myocardial infarction-like electrocardiographic changes in pregnant women, even if they do not have a history of hypertension.

## Background

Pheochromocytomas and paragangliomas (PPGLs) are neuroendocrine tumors of the adrenal medulla and paraganglia, respectively. PPGLs often secrete catecholamines, and an excess of catecholamines released by PPGLs may lead to hypertension, episodic sweating, palpitations, and headaches. PPGL patients can present with hypertensive or PPGL crises and may experience serious life-threatening cardiac and cerebrovascular complications [1]. The diagnosis of this disease is challenging due to significant heterogeneity in its clinical presentation. Hypertension is a common condition during pregnancy, and preeclampsia is the most common pregnancy-related complication. The ambiguous presentation of pheochromocytomas is frequently misinterpreted as preeclampsia. Although rare in pregnant women [2–4], pheochromocytomas pose a high risk of maternal and fetal death if not appropriately managed [5]. This case report describes a pregnant woman with a pheochromocytoma who underwent a cesarean section. The patient presented with sudden-onset chest pain, dyspnea, paroxysmal hypertension, and hypotension. It is uncommon for pregnant women with pheochromocytomas to develop cardiogenic shock after a cesarean section and to be successfully resuscitated using extracorporeal membrane oxygenation (ECMO) [6, 7].

## Case presentation

The patient was gravida 4, para 1 (G4P1). She delivered a baby by cesarean section when she was 28 years old, without any dangerous situations during delivery. The patient was a 32-year-old Chinese farmer at 31 weeks and three days of gestation with a history of gestational diabetes controlled by diet. The patient's blood pressure was 130/80 mmHg, and her blood glucose level was 7.0 mmol/L during pregnancy. The patient’s and her fetus’s vital signs were within normal limits. She was transferred to the emergency department due to persistent chest pain for one hour. The patient presented with typical angina symptoms, including retrosternal pressure-like pain located in the middle and lower part of the sternum and radiating pain from the back of the left shoulder, accompanied by chest tightness and sweating. However, she had no history of chest pain prior to this hospitalization. She had given birth several years prior and had no signs of gestational diabetes during pregnancy. She had no history of hypertension or other heart disease or a family history of hypertension, diabetes, heart disease or PPGLs. Her height was 1.7 m, her weight was 68.5 kg, and her body mass index (BMI) was 23.7 kg/m2. The patient's vital signs were within normal limits, including a blood pressure (BP) of 130/80 mmHg, a heart rate of 71 beats per minute (bpm), and an oral temperature of 36.3 °C. The lungs were not influenced upon auscultation, and the abdominal examination was unremarkable. No other abnormalities were found during the examination.

**Fig. 1:**
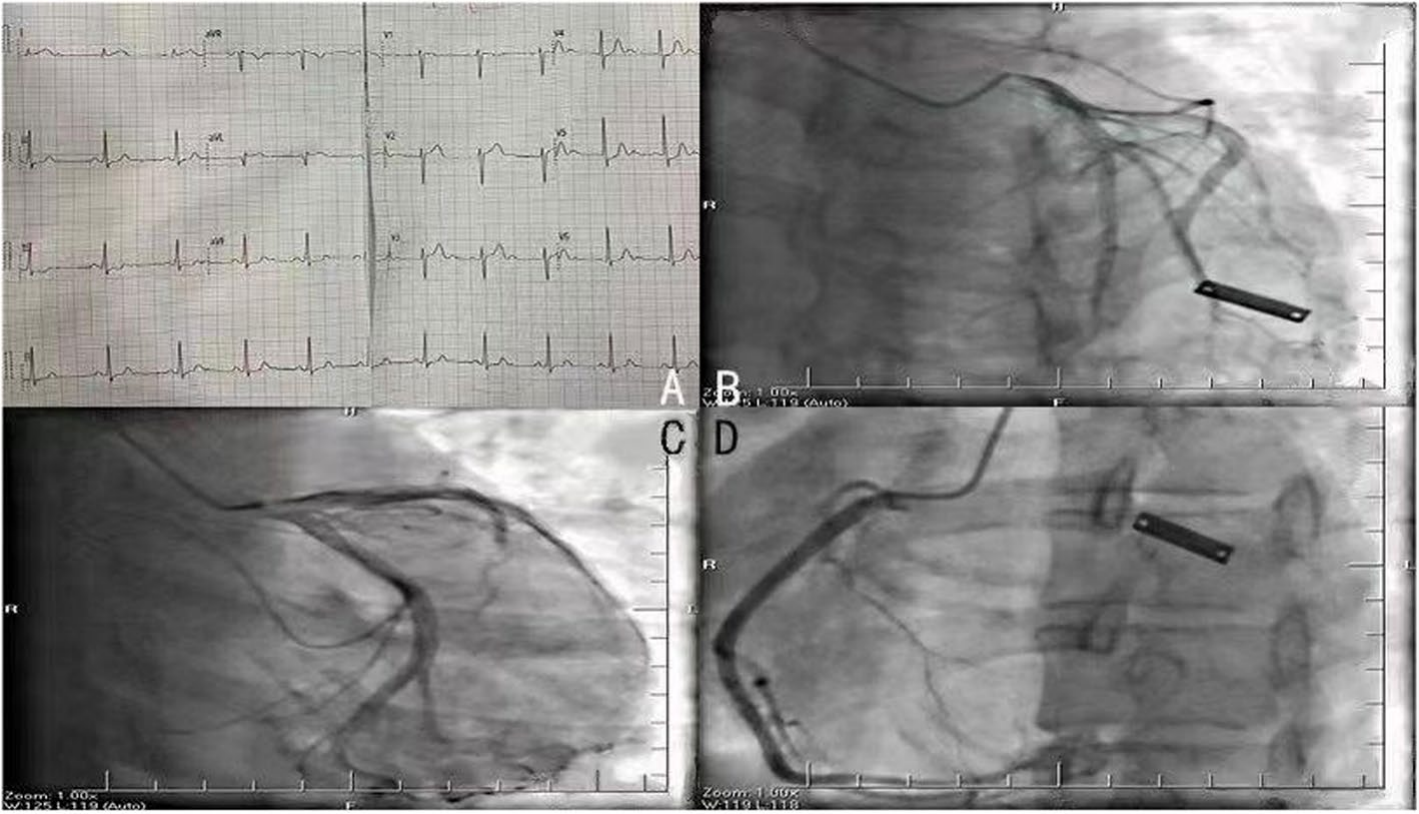
Electrocardiogram (**A**) and coronary angiography images of the left anterior descending branch (**B**), the coronary artery circumflex branch (**C**), and the right coronary artery (**D**)

**Fig. 2:**
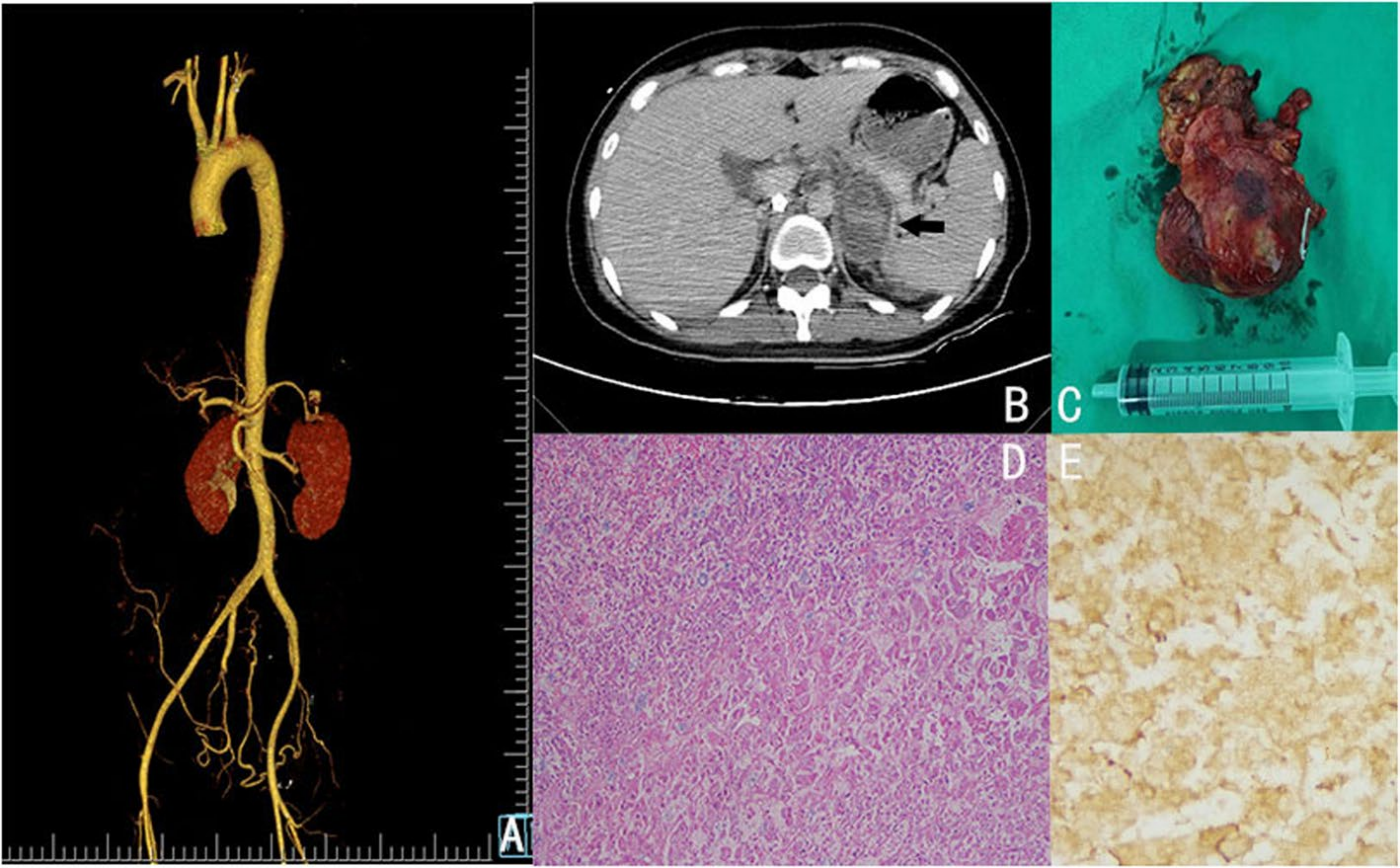
Computed tomography angiography of the thoracoabdominal aorta (**A**), a whole-abdominal enhanced CT scan revealing a cystic mass in the left adrenal region with hemorrhage (**B**, indicated by an arrow), a left-sided adrenal pheochromocytoma after resection (**C**), and pathological results (**D**, **E**)

The laboratory results were suggestive of normal renal function, liver dysfunction, hypokalemia, gestational diabetes, hyperlipidemia, and inflammation **(**Table 1). Electrocardiography (ECG) revealed sinus rhythm with acute high lateral wall ST-segment elevation in leads I and AVL (> 1 mm) and ST-segment depression in leads II, III, AVF, and V4-V6 (Fig. 1A). Her myoglobin level was 153 ng/ml (normal range, 9–82 ng/ml), and her cardiac troponin I level was 0.588 ng/ml (normal range, < 0.056 ng/ml). Transthoracic echocardiography revealed that the patient’s left ventricular systolic function (LVEF) was 60% and that she had no regional wall motion abnormalities, mild mitral regurgitation, and no significant pericardial cavity effusion. Upon re-examination, the patient’s cardiac enzyme levels presented slight elevation, accompanied by no further changes in ECG parameters. Abdominal ultrasound is crucial in the diagnosis of patients with suspected acute coronary syndrome to exclude acute pancreatitis, acute cholecystitis, gallbladder stones, and other acute abdominal conditions. We also conducted noninvasive tests, such as bedside abdominal aortic inferior vena cava ultrasound and bedside abdominal ultrasound. However, the cause of the patient's persistent chest pain could not be confirmed, so radiological and invasive tests were performed. The patient was administered 300 mg of aspirin, 180 mg of ticagrelor, and 10 mg of rosuvastatin. Coronary angiography revealed a normal coronary artery (Fig. 1B-D). Despite the procedures, the patient's chest pain persisted and could not be controlled. Therefore, thoracic aortic dissection was considered, and the three layers of the aorta had separated due to a disruption in the media layer, usually caused by intimal rupture, which results in bleeding in the aortic wall and acute chest pain. Computed tomography angiography (CTA) of the thoracoabdominal aorta was performed, and no vascular entrapment was detected (Fig. 2A).

**Table 1:** The laboratory results

Item	Results	Normal range
Alanine aminotransferase (U/L)	294	7–40
Aspartate aminotransferase (U/L)	223	7–40
Creatinine (umol/L)	66.4	40–130
Urea nitrogen (mmol/L)	5.1	2.3–7.1
Potassium (mmol/L)	3.2	3.5–5.3
Sodium (mmol/L)	141.8	135–147
Calcium (mmol/L)	2.37	1.9–2.5
Fasting glucose (mmol/L)	6.87	3.9–6.1
Low-density lipoprotein (mmol/L)	4.48	1.40–3.10
Triglycerides (mmol/L)	3.42	0.48–2.30
Leukocytes (/L)	17.6	4–10*10^9^
Erythrocytes (/L)	4.92	3.5–5.5*10^12^
Blood platelets (/L)	272	100–300*10^9^
Hemoglobin (g/L)	141	110–150

After being transferred to the ward, the patient developed new orthopnea despite receiving diuretic therapy and noninvasive ventilation. Her oxygen saturation level was 72% under breathing room air and 90% under supplemental oxygen. Arterial blood gas analysis revealed a pH of 7.293, an oxygen partial pressure of 78.1 mmHg, a CO_2_ partial pressure of 28.7 mmHg, and a lactic acid concentration of 6.4 mmol/L. Then, the patient received an intravenous infusion of 20 mg of furosemide and 90 mEq of sodium bicarbonate to correct the blood acid level.

Fetal heart rate monitoring revealed that the fetal heart rate ranged from 160 to 170 bpm, with a flat baseline and a poor response. An emergency cesarean delivery was performed under combined spinal-epidural anesthesia due to fetal distress. After birth, the newborn suffered from apnea, pale skin and a weak heartbeat, and had a weight of only 0.905 kg. The Apgar score was one at 1 min, six at 5 min, and six at 10 min. The operation lasted for 1.5 h. The patient's blood pressure was low during and after surgery, and she remained in shock after the procedure. Despite receiving high doses of vasoactive drugs such as milrinone, dobutamine, epinephrine, vasopressin, and norepinephrine, the patient remained hypotensive. Veno-arterial extracorporeal membrane oxygenationwas administered immediately.

The patient's vital signs remained stable even after pausing vasoactive drug use for one week. ECMO was subsequently withdrawn, and suturing of the right femoral artery was performed. During the operation, the patient's blood pressure significantly fluctuated, reaching a peak of 170/87 mmHg, accompanied by episodes of hypotension. Following the operation, the patient experienced persistent and intractable hypotension, with a minimum blood pressure of 35/21 mmHg. ECMO was restarted that night. Regarding the BP fluctuations, pheochromocytoma was considered, and biochemical tests revealed an elevated plasma metanephrine level of 12.98 nmol/L (normal range, ≤ 0.50 nmol/L) and normetanephrine level of 8.85 nmol/L (normal range, ≤ 0.90 nmol/L). The results of her 24-h urinary fractionated metanephrine and normetanephrine tests were as follows: metanephrine 2468 nmol/24 h (normal range, < 216 nmol/24 h) and normetanephrine 1120 nmol/24 h (normal range, < 312 nmol/24 h).

A subsequent CT scan of the abdomen revealed a wedge-shaped hypointense area in the middle of the left kidney, with mild enhancement in the delayed phase. Cystic occupancy with bleeding in the left adrenal region was also observed. **(**Fig. 2B). Based on these findings, left adrenal pheochromocytoma was suspected. On the 4th day after diagnosis, the patient underwent laparoscopic surgery on the left adrenal gland (Fig. 2C), and the pathological results confirmed the diagnosis of pheochromocytoma (Fig. 2D, E). The patient was discharged one week after laparoscopic surgery. In the short term, the patient’s condition improved significantly after the operation. Even after 1.5 months of follow-up, no abnormalities were detected in the patient, and she had a blood pressure of 104/66 mmHg, a fasting glucose level of 4.5 mmol/L (normal range, 3.9–6.1 mmol/L), and normal electrocardiographic and echocardiography results. After ten months of continuous monitoring, the patient's blood pressure remained within normal limits.

The newborn presented a poor response and respiratory distress and was immediately intubated with a tracheal tube and mechanically ventilated. The newborn had been diagnosed with hydrocephalus in the lateral ventricle at birth. During hospitalization, the infant received systematic supportive treatments such as alveolar surface active substances to improve lung compliance, anti-infection medication, and nutritional rehydration. Fresh frozen plasma was also infused to improve abnormal coagulation function, and a red blood cell suspension was infused to improve anemia symptoms. At the follow-up visit ten months after discharge from the hospital, the child's intelligence and physical development were also normal. However, even after several suggestions, the child’s parents declined genetic testing for pheochromocytoma.

## Discussion and conclusions

Neuroendocrine chromaffin cell tumors include pheochromocytomas and paragangliomas, which arise from chromaffin cells of the adrenal medulla (80%–85%) and are associated with the sympathetic ganglia (15%–20%) [2, 5]. They are rare catecholamine-producing tumors [3]. According to previous reports, pheochromocytomas and paragangliomas are rare during pregnancy. The reported incidence is 1/54,000 pregnancies [3]. Pheochromocytomas can secrete catecholamines such as norepinephrine, epinephrine, and dopamine, and a surge of catecholamines secreted by pheochromocytomas can cause severe cardiovascular consequences and even be fatal [3, 8]. In pregnant women with pheochromocytoma, ephemeral spiking of catecholamines, as well as uncontrolled hypertension, may disrupt placental circulation, leading to hypoxia, placental abruption or fetal demise [9]. Inappropriate diagnosis will result in maternal and fetal death [9]. Therefore, rapid and accurate diagnosis of this disease is critical.

The diagnosis of pheochromocytoma is challenging due to its varied clinical manifestations. In addition to various paroxysmal symptoms, such as hypertension, palpitations, sweating, and headache, excessive catecholamines can trigger cardiac arrhythmias, coronary spasms, myocardial infarction [1, 10], hemodynamic collapse, heart failure, pulmonary edema, hypotension, and cardiac arrest [4, 11–14]. Hypertension is often paroxysmal, as in our reported case; additionally, blood pressure may be unstable [2]. The common misconception is that hypertension during pregnancy is most likely due to gestational hypertension, and (pre)eclampsia often leads to a missed diagnosis of pheochromocytoma [3]. Our reported case suggests that when a pregnant woman presents with one of the mentioned acute severe cardiovascular symptoms, such as chest pain, regardless of whether the patient has high blood pressure, the diagnosis of potential pheochromocytoma should be considered [3]. Even with different modern diagnostic tests, the percentage of accurate prenatal diagnoses of pheochromocytomas is still between 70 and 75%, and many patients with pheochromocytomas are misdiagnosed before delivery [3, 15]. Initial laboratory evaluations are often based on clinical manifestations consistent with catecholamine excess [16]. High-risk patients should undergo 24-h urinary fractionated metanephrine and catecholamine or plasma fractionated metanephrine measurements [17–19]. Based on biochemical confirmation, a catecholamine tumor should also be considered, and the tumor needs to be localized by imaging, such as CT or magnetic resonance imaging (MRI) [3]. Once a pheochromocytoma is diagnosed and localized, alpha-blockade therapy should be used for at least ten days [20]. For this reason, we initially administered intravenous phentolamine to our patient. As soon as oral intake is feasible, phenoxybenzamine should be further administered. However, the main treatment for pheochromocytomas is surgical resection, which is often challenging due to the life-threatening risk of perioperative hemodynamic instability [21]. When the tumor is diagnosed in the second trimester of pregnancy, surgery to remove the pheochromocytoma should only be performed after adequate therapy with antiadrenergic agents [22]. In this reported case, considering that the patient’s hemodynamics were unstable, the patient underwent tumor removal surgery through left-sided adrenal laparoscopy on the fourth day after diagnosis and receptor blocker treatment.

This report describes the case of a pregnant woman with a pheochromocytoma, a rare condition in this population. The patient presented with angina pectoris resembling myocardial infarction despite no history of hypertension. This is an atypical clinical manifestation in patients with pheochromocytomas. Additionally, the electrocardiogram showed ST-segment elevation and elevated troponin levels. Coronary angiography and aortic CTA confirmed that there was no coronary artery disease or aortic dissection. Notably, this information was not readily available. It is rare for pregnant women with pheochromocytomas who develop cardiogenic shock after cesarean section surgery to be rescued by ECMO and successfully treated with short-term alpha-blocker therapy for surgical resection of the pheochromocytoma with ECMO support.

## Data Availability

The patient data for the present study are not publicly accessible due to local health research ethics protocols; however, deidentified data may be requested from the corresponding author.

## Abbreviations

PPGLs: Pheochromocytomas and paragangliomas
ECMO: Extracorporeal membrane oxygenation
BP: Blood pressure
Bpm: Beats per minute
ECG: Electrocardiogram
LVEF: Left ventricular systolic function
CT: Computed tomography
CTA: Computed tomography angiography
24 h: Twenty four hours
MRI: Magnetic resonance imaging
